# Combined Bottom-Up and Top-Down Approach for Highly Ordered One-Dimensional Composite Nanostructures for Spin Insulatronics

**DOI:** 10.1021/acsami.1c09582

**Published:** 2021-07-30

**Authors:** Gopal Datt, Ganesh Kotnana, Ramu Maddu, Örjan Vallin, Deep Chandra Joshi, Davide Peddis, Gianni Barucca, M. Venkata Kamalakar, Tapati Sarkar

**Affiliations:** † Department of Materials Science and Engineering, Uppsala University, Box 35, Uppsala SE-751 03, Sweden; ‡ Dipartimento di Chimica e Chimica Industriale, Università di Genova, Via Dodecaneso 31, Genova I-16146, Italy; § Institute of Structure of Matter, Italian National Research Council (CNR), Monterotondo Scalo, 00015 Rome, Italy; ∥ Department SIMAU, Università Politecnica delle Marche, Via Brecce Bianche 12, Ancona 60131, Italy; ⊥ Department of Physics and Astronomy, Uppsala University, Uppsala SE-751 20, Sweden

**Keywords:** one-dimensional nanostructures, bimagnetic nanocomposites, spin insulatronics, oxide electronics, strain coupling

## Abstract

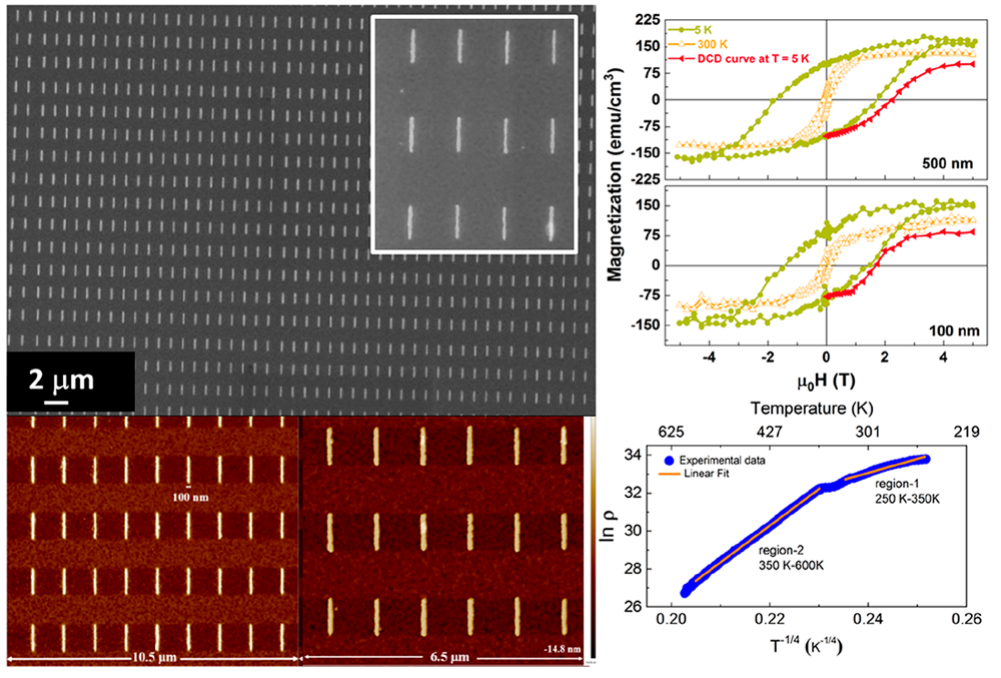

Engineering magnetic proximity effects-based devices requires developing efficient magnetic insulators. In particular, insulators, where magnetic phases show dramatic changes in texture on the nanometric level, could allow us to tune the proximity-induced exchange splitting at such distances. In this paper, we report the fabrication and characterization of highly ordered two-dimensional arrays of LaFeO_3_ (LFO)–CoFe_2_O_4_ (CFO) biphasic magnetic nanowires, grown on silicon substrates using a unique combination of bottom-up and top-down synthesis approaches. The regularity of the patterns was confirmed using atomic force microscopy and scanning electron microscopy techniques, whereas magnetic force microscopy images established the magnetic homogeneity of the patterned nanowires and absence of any magnetic debris between the wires. Transmission electron microscopy shows a close spatial correlation between the LFO and CFO phases, indicating strong grain-to-grain interfacial coupling, intrinsically different from the usual core–shell structures. Magnetic hysteresis loops reveal the ferrimagnetic nature of the composites up to room temperature and the presence of a strong magnetic coupling between the two phases, and electrical transport measurements demonstrate the strong insulating behavior of the LFO–CFO composite, which is found to be governed by Mott-variable range hopping conduction mechanisms. A shift in the Raman modes in the composite sample compared to those of pure CFO suggests the existence of strain-mediated elastic coupling between the two phases in the composite sample. Our work offers ordered composite nanowires with strong interfacial coupling between the two phases that can be directly integrated for developing multiphase spin insulatronic devices and emergent magnetic interfaces.

## 1 Introduction

Magnetic insulators^1^ are emerging materials for inducing novel attributes into spintronics, magnonics, and spin insulatronics,^2^ furthermore developing charge-neutral magnetic interfaces. A classic example is yttrium iron garnet,^3^ which has been studied for its low damping magnon transport, as well as for inducing proximity effects.^4^ More recent studies have shown that other classes of ferrimagnetic insulators, in particular, spinel ferrites, can offer advantages over garnets, for example, a less complex crystal structure, lower synthesis temperature, and better compatibility with other crystalline materials.^5^ On the other hand, today multifunctional composite materials^6^ are unique in this context, primarily because of their capability to incorporate the desired properties of the individual components. This results in a tailor-made end material that can be engineered for specific applications via introducing a wide variety of correlated phenomena. Magnetic oxides and their composites^7^ are thus being explored extensively, both with regard to making innovations in the synthesis techniques^8−12^ as well as efforts toward mimicking and/or improving on single-phase behavior in these biphasic compounds.^13−19^ Composite thin films and heterostructures have also been fabricated^20−29^ to broaden the prospects for applications.

In recent years, perovskite-spinel nanocomposite thin films^30^ and core–shell structures^9^ have attracted considerable attention. The interest lies in the flexibility of designing composite systems with a proper choice of the two component phases, their multifunctionality, and potential use in applications ranging from energy-efficient memory devices^31,32^ to creating artificial neurons and synapses for neuromorphic circuits.^33,34^ The insulating nature of many magnetic perovskite-spinel composite structures also makes them attractive for inducing proximity effects in nonmagnetic thin films. Composite systems where the individual components have intrinsically different magnetic orderings can be especially interesting to realize local variations in such proximity effects. However, such multifunctional systems have yet to find application in a real technological device, primarily due to the fact that they have been investigated mostly in the form of nanoparticles or large area films.^20^ Most device applications and device structures involve patterned nanostructures. Uniformity (in shape and size) as well as regularity of the patterns is essential for practical applications, as opposed to biphasic composites with one phase randomly located in a matrix of the second phase, which would make implementation in devices very difficult. In addition, maintaining the desired properties as obtained in particulate nanocomposites is necessary. The few reported attempts involving self-assembly approaches are either template-assisted or nucleation-induced, with one phase forming a regular pattern in an array of the second phase.^35,36^ Such patterns provide limited interfacial coupling between the two phases.

In this work, we report an innovative synthesis approach utilizing the advantages of both bottom-up and top-down components for fabricating highly ordered arrays of LaFeO_3_ (LFO)–CoFe_2_O_4_ (CFO) bimagnetic composite nanowires. LFO is a multiferroic system where the magnetic structure is a G-type antiferromagnet with a high ordering temperature of ∼750 K,^37^ and ferroelectric-like hysteresis loops have been observed at room temperature.^38^ In addition, LFO also shows a ferroelastic effect with coupling between the ferroelasticity and the antiferromagnetic spin orientation.^39^ CFO, on the other hand, is a ferrimagnet (ordering temperature ∼800 K) exhibiting high saturation magnetization (∼70–100 emu/g)^40,41^ and high anisotropy, with anisotropy constant values ranging between 1.8–3.0 × 10^6^ erg/cm^3^ (in bulk)^42^ that increases by an order of magnitude to 3.15 × 10^7^ erg/cm^3^ in nanoparticles.^43^ For CFO thin films, the magnetic properties are highly dependent on the degree of crystallization. The saturation magnetization (*M*_S_) and coercive fields (*H*_C_) of CFO thin films have been observed to increase with an increase in the degree of crystallization, with *M*_S_ = 110 emu/cm^3^ and out-of-plane *H*_C_ = 230 Oe in amorphous CFO film to *M*_S_ = 262 emu/cm^3^ and out-of-plane *H*_C_ = 4150 Oe in crystallized CFO thin film.^44^ Apart from the degree of crystallization, the crystal orientation also has a strong effect on the coercive fields, with a reported large out-of-plane coercivity of 11.3 kOe in (311)-preferred randomly oriented CFO thin film.^45^ Recently, a very high out-of-plane coercive field of 14.1 kOe and high saturation magnetization of 475 emu/cm^3^ has been reported in a preferentially oriented single-crystal-like textured thin film on an amorphous substrate grown using an innovative self-bilayer method.^46^

We have, thus, chosen CFO, with its ferrimagnetic properties (i.e., high saturation magnetization and moderate magnetic anisotropy), as a prototypical spinel ferrite and a viable choice of a ferrimagnetic insulator for spin insulatronics applications. Moreoever, among all spinel ferrites, CFO exhibits the highest magnetostriction value of ∼350 × 10^–6^.^42^ The incorporation of antiferromagnetic LFO as the second phase in the composite offers us a bimagnetic system, where the two phases have widely differing magnetic properties. Such a nanocomposite system with dramatic changes in magnetic texture at the nanometric level allows us to build a prototype for magnetic proximity effects-based devices using efficient magnetic insulators. Furthermore, the combination of ferroelastic LFO and magnetostrictive CFO offers us an excellent bimagnetic composite system that could enable the exploration of new functionalities for emergent technological applications. The biphasic LFO–CFO composite nanowires fabricated here show distinctly single-phase-like magnetic behavior. The magnetic coupling between the two phases has been explored using direct current demagnetization experiments. It is worth noting that although nanostructures of normal materials, sputtered films, e-beam evaporated materials, and multilayers can be readily prepared by lithographic techniques, the fabrication of composite nanostructures using the lithography process is intrinsically challenging because of the biphasic nature, and obtaining nanostructures very much depends upon the interfacial coupling between the individual phases. Careful optimizations have enabled us to create a patterned system that can be directly integrated into device structures, which is not possible for nanoparticles or large area films. In the following sections, we first present the details of the fabrication of the composite nanowires, followed by their structural, magnetic, and electrical transport characterization.

## 2 Synthesis and Experimental Techniques

### 2.1 Synthesis/Fabrication of LFO–CFO Nanowires

Here, we employ the concept of symbiotic synthesis of complex oxide nanocomposites,^47^ where both phases of the nanocomposite are formed at the same time to make the initial LFO–CFO composite. In a typical synthesis, two sols are first prepared by dissolving the respective precursors of LFO and CFO, which are then used to make a composite sol by mixing proportionate volumes of the LFO and CFO sols. The composite sol can be either heated to obtain a gel or spin-coated on Si substrates to form homogeneous composite films with both phases coexisting in the same layer.^29,47^ The precursors and solvents can be customized depending on the desired product (particulate composite or composite thin film). In the present case, we first obtained homogeneous LFO–CFO (50:50) bimagnetic composite thin films. Details of the thin film synthesis process are given in the Supporting Information. These composite thin films of thickness ∼50 nm were then used as base materials for fabricating LFO–CFO composite nanowires. The nanowires (with a width of 100 and 500 nm and aspect ratio 10:1) were fabricated via an electron beam lithography (EBL) patterning and etching process. For the patterning, 6% HSQ, a negative tone resist, was used for electron beam exposure. The exposed samples were then developed in a solution of AZ-326 followed by ion beam milling. Finally, any residue of HSQ or LFO/CFO films on the patterned structures were removed by dipping them in buffered HF solution. It is worth mentioning that such a lithographic process for composites is challenging and needs optimizations that we achieved here for a faithful pattern transfer. More details of the nanowire fabrication process along with a schematic flow chart (Figure S1) are given in the Supporting Information.

### 2.2 Characterization Techniques

The quality and regularity of the LFO–CFO nanowire patterns were investigated using a field emission scanning electron microscope (FESEM, Carl Zeiss, LEO1550 model) at an operating voltage of 3–5 kV. We also acquired atomic force microscopy (AFM) and magnetic force microscopy (MFM) images of the nanowires on a Bruker AFM Instrument, model Dimension Icon ICON4-SYS, at 300 K. The AFM imaging was done in tapping mode using an RTESP-300 AFM tip with a scan rate of 0.6/s. After the images were acquired, they were flattened with a line-by-line second-order fit to remove any artifacts/curvature imparted by the piezoelectric scanner. For recording of the magnetic phase images, a CoCr-coated silicon tip was used in a tapping-lift imaging mode at a lift height of 90 nm. Transmission electron microscopy (TEM, Philips CM200, operating at 200 kV) techniques were used to investigate the crystalline nature and quality of the fabricated nanowires. Samples for TEM analysis were prepared in a cross section using a conventional procedure consisting of cutting, in slices, the sample sandwiched between silicon substrates. Slices were prepared by grinding them on abrasive papers and diamond pastes. Disks with a 3 mm diameter were cut from the slices by an ultrasonic cutter (Gatan). To reduce the time of ion milling, in the last step of the mechanical thinning procedure, each 3 mm disk was mechanically thinned in a central area by a Dimple Grinder (Gatan). Final thinning was carried out by an ion beam system (Gatan PIPS) using Ar ions at 5 kV. Magnetic field-dependent magnetization of the nanowires was collected using a superconducting quantum interference device (SQUID) magnetometer from Quantum Design Inc. The samples were attached onto a piece of paper using GE Varnish (Oxford Instruments) and mounted with their planes parallel to the direction of the magnetic field. Magnetic hysteresis loops were recorded at *T* = 300 and 5 K in the −5 T to +5 T field range. At high fields, the diamagnetic contribution from the paper, varnish, and substrate starts dominating the *M*–*H* curves. To eliminate the diamagnetic contribution, we first estimated the diamagnetic susceptibility from the slope of the high-field data and then used this value to obtain the correct value of the magnetization using the equation: *M*_corr_ = *M*_exp_ – χ_dia_*H, where *M*_corr_ is the corrected value of the magnetization, *M*_exp_ is the experimentally measured value, χ_dia_ is the slope of the high-field region, and *H* is the magnetic field. A temperature-dependent resistivity measurement was performed using a Keithley 6221 current source and Keithley 2182A nanovoltmeter coupled with a customized liquid-nitrogen-based cryostat from Janis. The temperature of the cryostat was controlled using a Lakeshore-335 temperature controller. Electrical contacts (four-probe method) were prepared using silver paint on a rectangular thin film. A constant current was applied using a Keithley 6221 current source and the potential difference was measured by the nanovoltmeter. The Raman spectra of the samples were recorded with Renishaw-Micro-Raman spectroscopy using a He–Ne laser with an excitation wavelength of 532 nm. To avoid any overheating of the samples during the measurement, the power of the laser was kept below 1 mW.

## 3 Results and Discussion

### 3.1 Regularity and Quality of the Patterns

**Figure 1:**
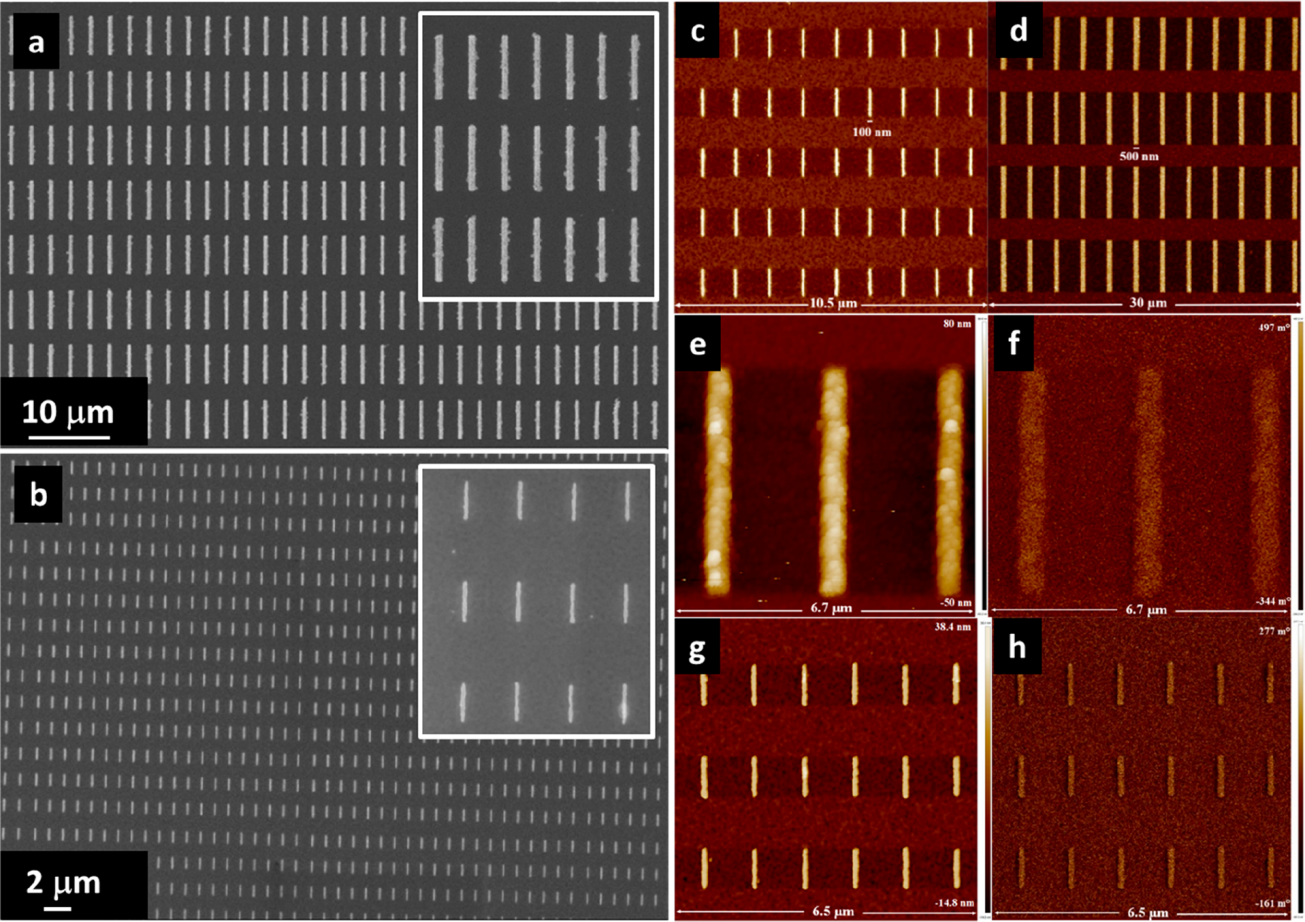
LFO–CFO composite nanowires: FESEM micrographs of nanowires of width (a) 500 nm and (b) 100 nm. The corresponding insets show enlarged views of the nanowires. AFM images of composite nanowires of width (c) 100 nm and (d) 500 nm. MFM (e) topographic and (f) phase image of composite nanowires of width 500 nm. MFM (g) topographic and (h) phase image of composite nanowires of width 100 nm.

The quality and regularity of the patterns were checked over large areas using FESEM and AFM. Figure 1a,b shows the FESEM micrographs of both 500 nm (Figure 1a) and 100 nm (Figure 1b) width nanowires. A typical image taken on a larger area is shown in the Supporting Information (Figure S2). It can be seen from the FESEM micrographs that both the 100 and 500 nm width nanowires are of well-defined shape over a large area and no defects are present between the nanowires. The FESEM micrographs confirm the high pattern fidelity along with good control over the shape and size of the fabricated nanowires.

AFM images of the samples are shown in Figure 1c,d. They further confirm that the obtained nanowires follow the desired patterns as per the lithographic patterning process, creating perfectly regular structures over large areas. It can also be observed from the AFM micrographs that no debris or artifacts are present between the nanowires, which may otherwise affect the magnetic properties. The local magnetization (magnetic microstructure) of these nanowires was investigated using MFM, and the obtained topographic and phase images are depicted in Figure 1e–h. Figure 1e shows the topographic image of LFO–CFO wires of width 500 nm, and Figure 1f is the corresponding phase image. The latter, in particular, reveals that the wires are strongly magnetic in nature and the magnetization is homogeneous across their length. Figure 1g,h shows the topographic and magnetic phase images, respectively, of the 100 nm width wires. It is observed from the MFM phase image that these wires are homogeneously magnetic across the length, though the contrast of the stray magnetic field is weaker than that of the 500 nm width wires. The weakening of the stray magnetic field can be ascribed to the significant reduction of ferrimagnetic materials volume in the 100 nm wires. Furthermore, the magnetic phase images of both nanowire patterns indicate that no magnetic residuals from the LFO–CFO film are left over between the wires (as no dark/bright area of stray field is observed between the nanowire structures). The AFM and MFM images, therefore, confirm that the fabricated nanowires are of good quality and magnetically homogeneous over their entire lengths.

### 3.2 Internal Morphology of the Composite Nanowires

**Figure 2:**
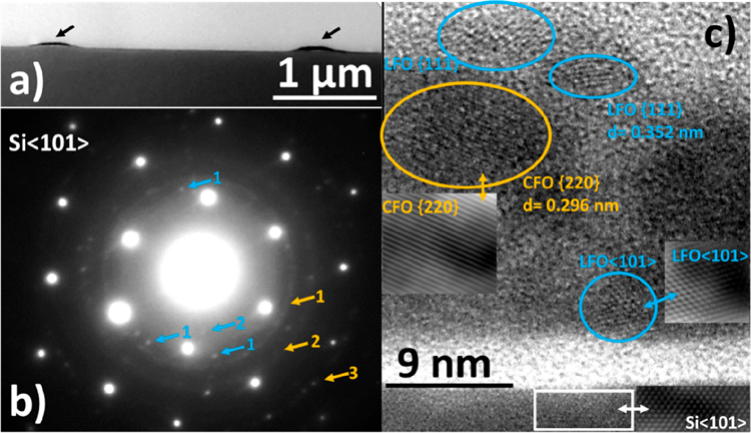
(a) Cross-sectional bright field TEM image showing two LFO–CFO 500 nm width nanowires; (b) SAED pattern obtained from one of the LFO–CFO nanowires; (c) HR-TEM image of an LFO–CFO nanowire.

To investigate in more detail the structure of the composite nanowires, transmission electron microscopy (TEM) techniques were applied on cross-sectioned samples cut perpendicularly to the nanowires’ length. In particular, Figure 2a shows a bright field TEM image of two nanowires (black arrows) visible along the short side. The nanowires are well-defined over the Si substrate without the presence of residual LFO–CFO film between them. Observations performed in other areas of the sample confirm the good separation and definition of the nanowires, thus revealing the effectiveness of the proposed EBL process for patterning LFO–CFO composite nanowires on LFO–CFO composite thin films. The phase composition and crystalline nature of the nanowires were investigated by selected area electron diffraction (SAED) measurements. The SAED pattern of the nanowire shown in the left side of Figure 2a is imaged in Figure 2b. The most intense diffraction spots, regularly distributed, are due to the Si substrate oriented in the ⟨101⟩ zone axis. In addition to these, it is possible to observe the presence of other feeble diffraction spots, randomly oriented or arranged in circles, which can be attributed to the presence of the LFO (blue arrows: experimental interplanar distances *d*(1) = (0.396 ± 0.004) nm and *d*(2) = (0.279 ± 0.003) nm attributed to *d*_LFO_(101) = 0.393015 nm and *d*_LFO_(121) = 0.277855 nm, International Centre for Diffraction Data (ICDD) card no. 37-1493) and CFO (orange arrows: experimental values *d*(1) = (0.256 ± 0.003) nm, *d*(2) = (0.209 ± 0.002) nm, and *d*(3) = (0.149 ± 0.002) nm attributed to *d*_CFO_(311) = 0.253100 nm, *d*_CFO_(400) = 0.209900 nm, and *d*_CFO_(440) = 0.148300 nm, ICDD-card no. 22-1086) phases, respectively. The distribution of the two phases inside the nanowires was investigated by high-resolution (HR) TEM analysis. A typical HR-TEM image of a portion of a 500 nm width nanowire is shown in Figure 2c. Some fringes (blue and orange circles) due to the presence of crystalline nanoparticles in good orientation with respect to the electron beam in the microscope are clearly visible. The image has been investigated by fast Fourier transform (FFT) analysis, allowing precise determination of the lattice interplanar distances associated with the visible fringes and to identify the CFO and LFO phases. The obtained families of atomic planes, some measured interplanar distances, and the phases are reported in the image together with three insets showing the masked inverse FFTs of the corresponding areas of the sample and the Si substrate. The procedure consists of the FFT of the image, the application of a mask to remove the noise, and an inverse FFT to reobtain the image. The atomic periodicities of the corresponding crystalline regions are clearly evidenced in this way. HR-TEM observations establish that the LFO and CFO phases are highly crystalline in nature and homogeneously distributed across the nanowires. It is important to note here that interdiffusion of cations at the interface often plays an important role in composites, giving unprecedented access to new physics and effects emerging at oxide interfaces.^48^ Rietveld analysis on X-ray diffraction patterns on our composite samples suggests that that there is no appreciable interdiffusion of the cations into the bulk of the nanocrystals (see Figure S3 and related text in the Supporting Information).

### 3.3 Strongly Coupled Composite Nanowires

The magnetic ordering temperatures of both phases (LFO and CFO) in the composite samples occur much above room temperature. The maximum accessible temperature of 400 K using our SQUID magnetometer (that is much below the magnetic ordering temperatures of the two phases) put a constraint on probing the ordering temperatures via magnetization versus temperature measurements. Nevertheless, the recorded isothermal magnetization curves and direct current demagnetization experiments provide key information about the magnetic properties of the samples.

**Figure 3:**
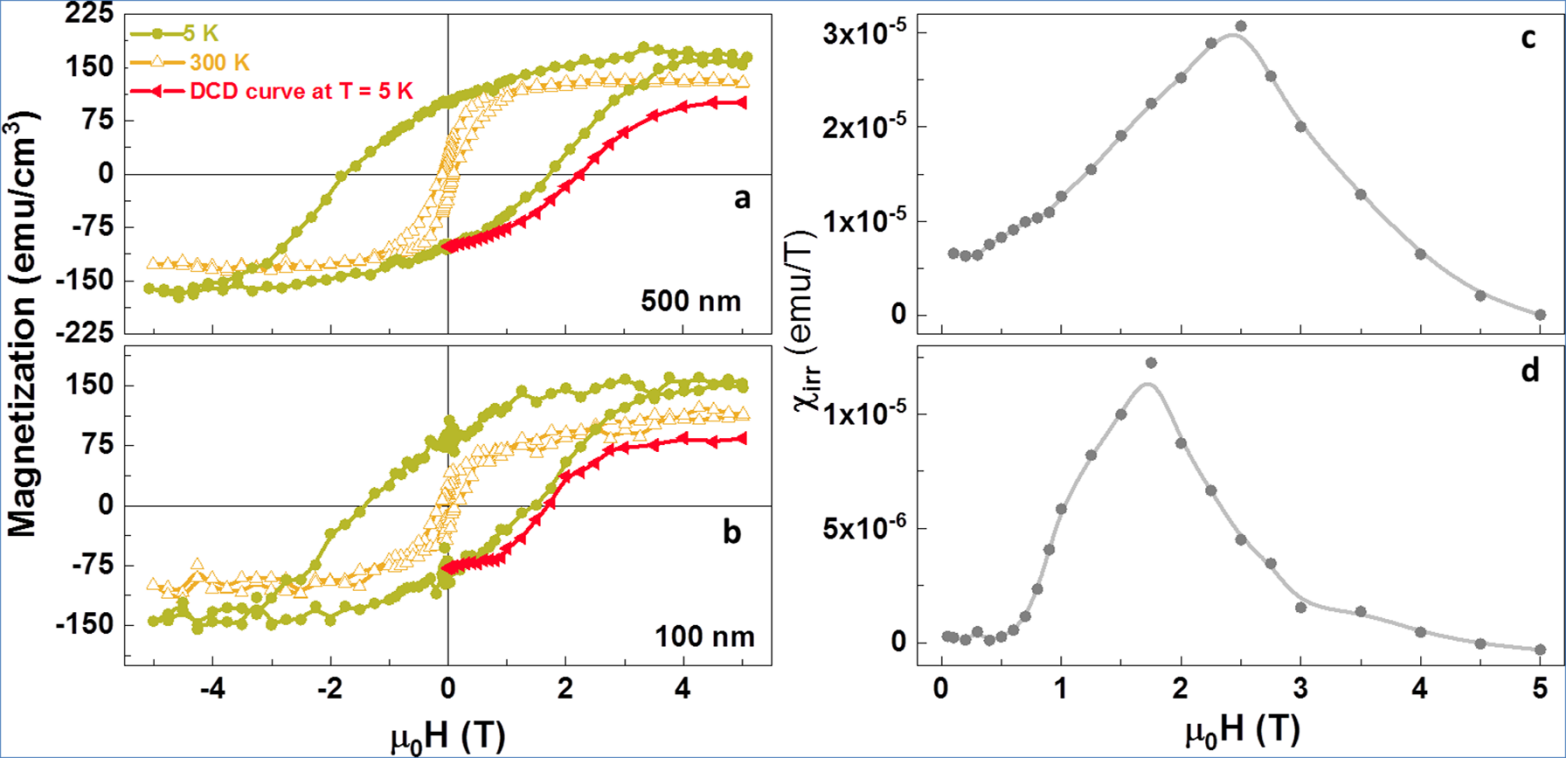
Isothermal magnetization curves at *T* = 5 and 300 K, and *M*_DCD_ versus a reverse magnetic field for LFO–CFO composite nanowires of width (a) 500 nm and (b) 100 nm. (c) and (d) show the respective switching field distributions (for details, see text).

In Figure 3, we summarize the magnetic properties of the composite nanowires. The isothermal magnetization curves recorded at 5 and 300 K (Figure 3a,b) reveal that the nanowires are ferrimagnetic in nature with open hysteresis loops reminiscent of that of pure CFO.^40^ Despite the presence of 50% antiferromagnetic LFO, the ferrimagnetic nature with an open hysteresis loop is maintained until 300 K in the 50/50 LFO/CFO composite nanowires. For comparison, we have also shown the isothermal magnetization curves of the LFO–CFO composite film (before patterning) in the Supporting Information (Figure S4). In Table 1, we summarize the saturation magnetization and coercivity values of our composite nanowire and thin film samples, as well as compare them with corresponding reported values in CFO thin films. Considering the 50% phase fraction of CFO in our composite samples, the saturation magnetization values match well with the reported values of *M*_S_ in polycrystalline CFO thin films,^44,45^ though they are smaller than the value of *M*_S_ for a single-crystal-like CFO thin film.^46^ The room-temperature coercive field values of the composite samples are smaller than that of CFO thin films because of the effect of the 50% phase fraction of antiferromagnetic LFO in our samples. The extremely low magnetic moment of the LFO phase was below the resolution limit of the SQUID magnetometer, which put a limitation on obtaining *M*–*H* curves of pure LFO samples (without the CFO phase).

**Table 1:** Comparison of Room-Temperature Saturation Magnetization and Coercivity Values in Composite Nanowire and Thin Film Samples

sample	saturation magnetization (emu/cm^3^)	in-plane coercive field (T)
LFO–CFO nanowire (100 nm width)	122 (4)	0.140 (4)
LFO–CFO nanowire (500 nm width)	135 (4)	0.090 (3)
LFO–CFO film	186 (6)	0.200 (6)
crystallized CFO film^44^	262 (8)	0.270 (8)
(311)-preferred randomly oriented CFO film^45^	225 (7)	0.47 (1)
single-crystal-like CFO thin film^46^	475 (14)	0.50 (2)

Exchange bias measurements performed on the composite samples did not yield any observable shift of the *M*–*H* curves (Figure S5 in the Supporting Information). The absence of exchange bias could arise from a modification of the antiferromagnetic ordering strength in the nanocrystals of LaFeO_3_ in the composite sample. For exchange bias to occur, the antiferromagnetic anisotropy should be sufficiently large so that the spins of the antiferromagnetic phase remain unchanged and can pin the spins of the ferromagnetic phase. Nanoparticles of LaFeO_3_ have been observed to be weakly ferromagnetic.^49^ Because the extremely low magnetic moment of our single-phase LFO thin film (without the CFO phase) was below the resolution limit of the SQUID magnetometer, we probed the room-temperature (300 K) *M*–*H* curve of LFO nanocrystals synthesized via sol–gel synthesis using the same precursors as used for the film synthesis. The LFO nanocrystals show nonsaturating behavior (Figure S6 in the Supporting Information), indicating their room-temperature antiferromagnetic nature. However, a close look at the low-field values (inset of Figure S6) clearly shows an open loop with a small coercive field of ∼0.01 T. This is indicative of a weak ferromagnetic phase, as has been observed previously in nanosized LFO.^49^ This suggests that the LFO phase in the thin film (consisting of similar nanocrystals) is antiferromagnetic, but has a weak ferromagnetic component arising from uncompensated spins at the surfaces of the nanocrystals. Thus, our nanocomposite system has uncompensated spins at the interface between the ferromagnet and antiferromagnet. If the uncompensated spins can rotate with the ferromagnet, there is no observable exchange bias. Such rotatable uncompensated spins of the antiferromagnetic (or weakly ferromagnetic) phase have been observed before in BiFeO_3_–CoFe_2_O_4_ nanostructures.^50^ Thus, a weak ferromagnetic ordering or an antiferromagnetic ordering with a modified antiferromagnetic anisotropy due to size reduction could lead to an absence of the exchange bias effect because of the inability of the spins to pin the ferromagnetic layer.

In such a case, where exchange bias measurements do not give much information, it is worthwhile to probe the magnetic coupling between the two phases using other methods. To probe the extent of magnetic coupling in composite materials, direct current demagnetization (DCD) experiments have been established to be a quick, easy, and reliable way.^17,19,51−53^ Earlier, we performed DCD experiments on continuous composite films and found a strong coupling between the two magnetic phases in such films.^29^ However, because the nanowire samples were fabricated using a top-down approach, it is necessary to check the quality (in particular, the coupling between the two magnetic phases) postfabrication. The DCD curves of the 500 and 100 nm width composite wires acquired at 5 K are also shown in (a) and (b), respecitvely (red curves), of Figure 3. These curves were obtained by first saturating the sample in a negative field (−5 T) and then measuring the remanent magnetization after applying and switching off reverse (positive) fields of increasing amplitude up to *H* = +5 T, thereby investigating the irreversible process of magnetization in the samples. The differentiated curve of *M*_DCD_ with respect to H gives the irreversible component of susceptibility, χ_irr_ (Figure 3c,d). χ_irr_ is a measure of the energy barrier distribution, which is associated with the distribution of the switching field, defined as the field necessary to overcome the energy barrier during an irreversible process.^52,54^ For a bimagnetic composite system with weak coupling between the two magnetic phases, the χ_irr_ plots show two distinct peaks corresponding to the reversal processes of the two individual phases, quite independent of each other;^12,19,55^ see also Figure S7 in the Supporting Information. The composite nanowires, however, are clearly characterized by a single peak, even if quite broad, in the χ_irr_ versus magnetic field plots, suggesting a single average switching field despite the presence of two magnetic phases, which have widely differing switching fields individually (individual switching fields of LFO and CFO differ by more than an order of magnitude^18^). This confirms that the two phases in the LFO–CFO composite nanowires are strongly coupled magnetically, the source of which can be attributed to the simultaneous biphasic synthesis method that ensures the maximum possible interfacial contact between the two phases.^56^ These experiments ensure that the composite nanowires retain the strong magnetic coupling between the two phases, even after being subjected to e-beam lithography, and that there is no detrimental effect caused by the lithography process.

The magnetic coupling between the two phases can be either exchange-coupled or dipolar in origin. Dipolar forces are long-range, that is, particles that are not in contact with each other can interact via long-range dipolar forces, whereas exchange coupling becomes more important for the particles that are in contact with each other. Because the simultaneous biphasic growth method that we use to fabricate the LFO–CFO composite film allows a closer spatial correlation between the two phases, it is expected that the exchange coupling plays a stronger role.

### 3.4 Insulating Character of the LFO–CFO Composite

**Figure 4:**
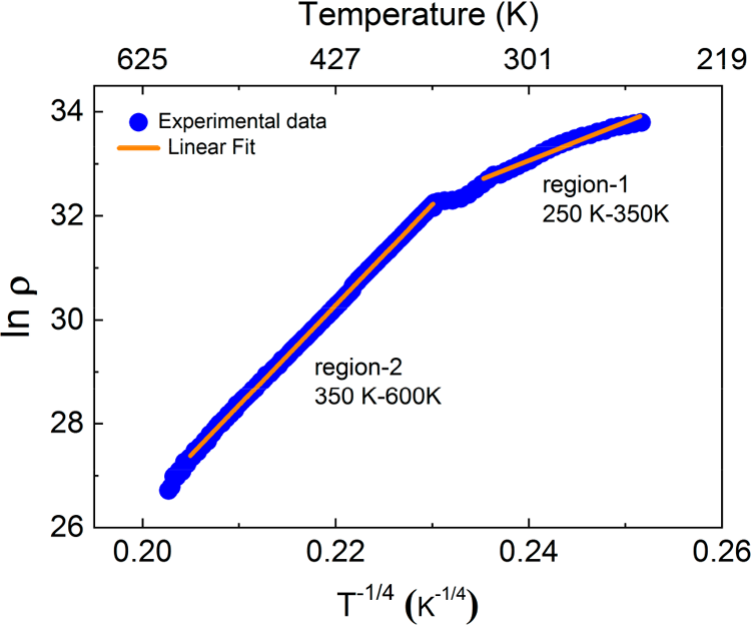
Temperature variation of resistivity of LFO–CFO film with a linear fit of ln ρ vs *T*^–1/4^. Two different slope regions are evident in the plot.

To characterize the electrical nature of our samples, we performed temperature-dependent resistivity measurements. We found that the LFO–CFO composite wires were highly insulating with resistance values higher than the measurement range of our setup. To circumvent this, we measured the resistivity of the parent LFO–CFO composite film (without patterning) in the temperature range 250–600 K (Figure 4). At room temperature, the resistivity was found be ∼2 × 10^14^ Ω-cm, which increased further below room temperature. It has been reported previously that the transport mechanism of single-phase ferrites^57^ as well as ferrite-based composites^58^ can be explained using the variable range hopping (VRH) of the localized carriers. According to the VRH model, the resistivity follows the generalized equation ρ(*T*) = ρ_0_*T*^*p*^ exp[(*T*_0_/*T*)^*q*^], where ρ_0_ is a prefactor and *T*_0_ is a characteristic temperature.^59^ The factors *p* and *q* depend on the system’s dimensionality and the variation of the density of states, *N*(*E*_F_), near the Fermi energy level, respectively. For *p* = 0 and *q* = ^1^/_4_, one gets the Motts-VRH model, where *N*(*E*_F_) remains almost constant near the Fermi energy level.^60^ In contrast, for *p* = *q* = ^1^/_2_, one gets the Efros-Shklovskii (ES) model, in which there occurs a Coulomb gap (vanishing density of states) in *N*(*E*_F_) near the Fermi energy level due to strong Coulomb interaction between the charge carriers.^60^ To investigate which model explains our experimental results better, we used both models to fit the resistivity data. The linear fit for the plot between ln ρ versus *T*^–1/4^ was found to attain the least value of the residual sum of squares error,^61^ which suggests that the Mott-VRH model governs the transport properties of the LFO–CFO composite films with a three-dimensional charge transport mechanism.

The Mott-VRH equation can be written as^60^
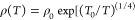
1where the characteristic Mott temperature *T*_0_ = *a*/[*k*_B_*N*(*E*_F_)ξ^3^]; *N*(*E*_F_) is the density of the localized state at the Fermi level, *k*_B_ is the Boltzmann constant, ξ is the decay length of the localized polaron wave function, and *a* is a constant with a value of 64.^59^ From the value of the characteristic Mott temperature *T*_0_, the mean hopping distance (*R*_hop_) and the hopping energy, Δ_hop_, can be calculated using the following relations^62^
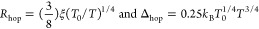
2The fit of the experimental resistivity data using the Mott-VRH equation (1) is also shown in Figure 4. It is clear from Figure 4 that the plot shows two different slopes, one in a low-temperature region (250–350 K) and the other in a high-temperature region (350–600 K). Therefore, to obtain the correct values of the different parameters, both regions were fitted separately. *T*_0_ was obtained from the slope of the linear fit, whereas *N*(*E*_F_*)*, *R*_hop_, and Δ_hop_ were calculated at *T* = 300 and 500 K using the obtained value of *T*_0_. All the obtained parameters are listed in Table 2.

**Table 2:** Different Parameters Obtained by Fitting the Resistivity Data with the Mott-VRH Model

	VRH exponent ^1^/_4_			
temperature range	*T*_0_ (K) × 10^7^	*N*(*E*_F_) (eV^–1^ cm^–3^)	*R*_hop_ (Å)	Δ_hop_ (eV)
250–350 K	2.9 (2)	4.4 (2) × 10^19^	36.3 (5)	8.2 (1)
350–600 K	140.0 (1)	9.14 (2) × 10^17^	83.8 (5)	46.57 (3)

The characteristic temperature, *T*_0_, is found to increase with temperature, and consequently, the density of the localized state, *N*(*E*_F_), decreases. In the low-temperature regime (250–350 K), the obtained value of *T*_0_ matches well with the reported values in epitaxial spinel ferrite thin films grown using pulsed laser deposition, for example, *T*_0_ = 1.4–9.8 × 10^7^ K and 5.1–8.0 × 10^7^ K in Ru-substituted CFO thin films^63^ and Co-substituted Fe_3_O_4_ thin films,^64^ respectively. Interestingly, though, in the high-temperature region (350–600 K), the obtained value of *T*_0_ is found to be closer to that of yttrium iron garnet (∼10^9^–10^10^ K).^65^ From the above analysis, it is concluded that the strongly insulating transport mechanism of the LFO–CFO composite system is governed by the Mott-VRH hopping model. This makes the LFO–CFO composite thin films and the possibility to prepare composite nanowires from such thin films a significant step forward in engineering novel magnetic proximity effects-based devices using efficient magnetic insulators. The sharp changes in the magnetic texture at the nanometric level in such composites could allow for sharp changes in the exchange splitting for developing novel magnetoresistive and magnonic devices. It is worth noting that the method demonstrated here is cost-effective when compared to conventional thin film deposition methods (viz., pulsed laser deposition, molecular beam epitaxy, and magnetron sputtering). The use of this versatile technique could be further extended to fabricate highly ordered patterns of other binary complex oxides/two-phase multiferroic and anisotropic systems with precise control over shape, size, and composition. This makes our work an important step forward in the fabrication of well-defined patterns of multifunctional composite nanostructures for advanced technological applications.

### 3.5 Strain Coupling in the LFO–CFO Composite

**Figure 5:**
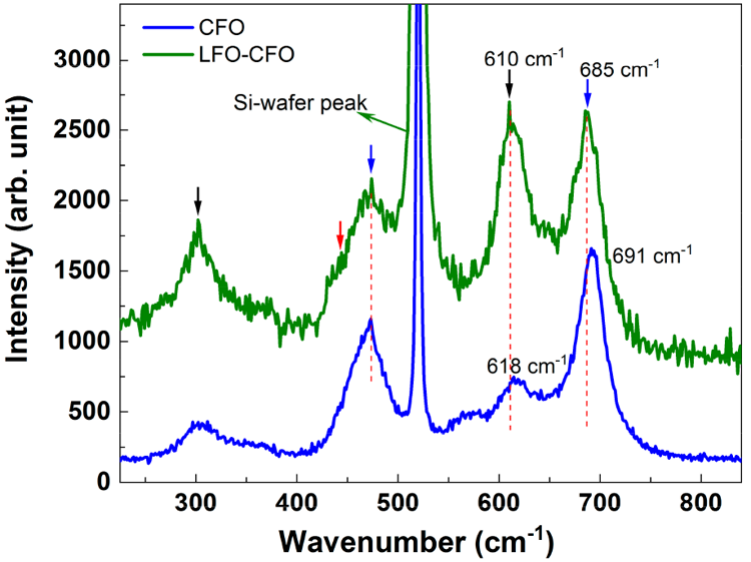
Raman spectra of CFO and LFO–CFO samples (for details, see text).

Raman spectroscopy is an efficient probe to investigate the strain/stress-induced coupling in composite systems because it is very sensitive to any type of localized structural changes/distortion induced by external strain/stress. It has been extensively used to investigate and establish the coupling in cobalt ferrite-based composite systems.^66,67^ Therefore, to investigate the strain coupling between the LFO and CFO phases in our samples, we recorded the Raman spectra of the CFO and LFO–CFO samples at 300 K in the range of 200–900 cm^–1^ (Figure 5).

The Raman spectra of the CFO sample exhibits four major Raman peaks corresponding to the *Fd*3̅*m* spinel structure of CFO.^68^ The observed peak positions and corresponding polyhedral are presented in Table 3. The Raman peaks in the range of 550–720 cm^–1^ correspond to the A_1g_ mode, which appears due to the vibration of the Fe–O_4_/Co–O_4_ tetrahedra, whereas the peaks appearing below 500 cm^–1^ are due to the E_g_ and T_2g_ vibrations of the Fe–O_6_/Co–O_6_ octahedra. On the other hand, LFO has an orthorhombic *Pnma*-type structure, and it is reported to exhibit Raman modes between 400 and 450 cm^–1^ because of the bending vibration of the Fe–O_6_, whereas the mode corresponding to the stretching vibration of the Fe–O_6_ appears above 500 cm^–1^.^69,70^ Raman peaks corresponding to the A_1g_ vibration of the rare-earth ion (La) are reported to be observed below 300 cm^–1^.

**Table 3:** Observed Peak Positions in the Raman Spectra along with the Corresponding Modes

polyhedra	Raman mode	peak position in CFO sample	peak position in composite sample
FeO_6_ octa	E_g_	302 (1) cm^–1^	302 (1) cm^–1^
FeO_6_/CoO_6_	T_2g_	473 (1) cm^–1^	473 (1) cm^–1^
Fe–O_4_ tetra	A_1g_	618 (1) cm^–1^	610 (1) cm^–1^
Co/Fe–O_4_ tetra	A_1g_	691 (1) cm^–1^	685 (1) cm^–1^

The presence of strain in composite samples is seen as a shift in the Raman modes, wherein compressive (tensile) strain causes the shift in wave number toward the higher (lower) side.^66^ Therefore, to investigate the presence of strain-mediated elastic interaction between the two phases of the composite sample, we recorded the Raman spectra of the composite LFO–CFO sample and compared it to the Raman spectra of the CFO sample in Figure 5.

In the Raman spectra for the LFO–CFO sample, the peaks corresponding to both LFO and CFO phases (610 and 300 cm^–1^), only the CFO phase (473 and 685 cm^–1^), and only the LFO phase (small shoulder near 430 cm^–1^) are marked in Figure 5 by black, blue, and red arrows, respectively. We observe that the peaks at 691 and 618 cm^–1^ that correspond to the tetrahedra vibrations of the A_1g_ mode of CFO exhibit clear shifts in the composite peak toward a lower wave number (by 6 and 8 cm^–1^, respectively). This observed shift in the A_1g_ Raman mode of the LFO–CFO composite sample indicates that there exists an elastic interaction between the two phases in the composite.^66^

The A_1g_ Raman mode near 690 cm^–1^ is reported to arise because of the stretching vibration of Fe–O_4_ tetrahedra along the {111}-direction of the cubic spinel structure.^68^ Iliev et al. proposed that this Fe–O_4_ bond elongates with the elongation of the {111}-space diagonal *d*; consequently, the A_1g_ Raman mode frequency decreases with increasing *d* and vice versa.^68^ As compressive (tensile) strain causes the shift in wave number toward the higher (lower) side,^66^ therefore, the shift of the A_1g_ Raman mode in the LFO–CFO film toward a lower wave number indicates that the CFO phase exhibits an out-of-plane compressive strain in the {111} direction. Therefore, from the above Raman analysis, it can be concluded that there exists a strong strain-mediated elastic interaction between the two phases of the composite. This fulfills a key necessary condition for the presence of magneto-electric coupling in these systems because magneto-electric coupling in composite systems originates from strain coupling. The presence of strain and elastic interaction between the two phases, as established from our Raman results, suggest that these composite systems are potential candidates for exhibiting magneto-electric effects, which will be studied in the future.

## 4 Conclusions

In summary, we demonstrated a novel approach for the fabrication of biphasic magnetic nanowires of LFO–CFO insulating nanocomposites by combining top-down and bottom-up approaches. Patterns of LFO–CFO nanowires down to 100 nm have been fabricated, which show well-defined shapes and sizes with no artifacts/residues of magnetic film present between the nanowires. The homogeneous composite nanowires exhibit a close spatial correlation between the two phases of the composite. The field dependence of magnetization reveals room-temperature ferrimagnetic properties of the nanowires and the presence of strong magnetic coupling between the two phases. Electrical transport measurements on the composite display the strong insulating behavior of LFO–CFO governed by Mott-VRH conduction mechanisms, and with a high room-temperature resistivity value of ∼10^14^ Ω-cm. Furthermore, the existence of strain coupling in the composite has been explored using Raman spectroscopy, revealing a strong strain-mediated elastic interaction between the two phases of the composite. Our work provides a pathway for developing complex oxide multiphase magnetic insulators and their nanostructures that can be of significance for advanced devices and new magnonic sensors designed by sharply varying proximity effects.

## Supporting Information Available

Details of the LFO–CFO composite thin film synthesis process and nanowire fabrication process, FESEM image of a large patterned area, isothermal magnetization curves of LFO–CFO composite thin film, isothermal magnetization curves of LFO–CFO composite nanowire recorded after zero field cooling and field cooling, isothermal magnetization curve of LFO nanocrystals, and switching field distributions of weakly coupled LFO–CFO particulate nanocomposites (PDF)
