# Supplementary material for: Combined Bottom-Up and Top-Down Approach for Highly Ordered One-Dimensional Composite Nanostructures for Spin Insulatronics
Source: ACS Appl Mater Interfaces. 2021 Jul 30;13(31):37500–9. doi: 10.1021/acsami.1c09582 (PMC8397244; doi:10.1021/acsami.1c09582)
Supplement: Supplementary file 1 — am1c09582_si_001.pdf [file am1c09582_si_001.pdf]

Electronic Supplementary Information for

Combined bottom-up and top-down approach for highly-ordered one-dimensional composite nanostructures for spin insulatronics

Gopal Datt[1], Ganesh Kotnana[1], Ramu Maddu[1], Örjan Vallin[1], Deep Chandra Joshi[1], Davide Peddis[2,3], Gianni Barucca[4], M. Venkata Kamalakar[5], and Tapati Sarkar[1†]*

[1]Department of Materials Science and Engineering, Uppsala University, Box 35, SE-751 03, Uppsala, Sweden
[2]Dipartimento di Chimica e Chimica Industriale, Università di Genova, Via Dodecaneso 31, I-16146, Genova, Italy
[3]Institute of Structure of Matter, Italian National Research Council (CNR), 00015 Monterotondo Scalo, Rome, Italy
[4]Department SIMAU, Università Politecnica delle Marche, Via Brecce Bianche 12, 60131 Ancona, Italy
[5]Department of Physics and Astronomy, Uppsala University, SE-751 20, Uppsala, Sweden

* venkata.mutta@physics.uu.se
† tapati.sarkar@angstrom.uu.se

Synthesis of LFO-CFO composite thin film

For the synthesis of single-layer biphasic LFO-CFO composite thin film, cobalt acetate tetrahydrate, iron nitrate nonahydrate, and lanthanum nitrate hexahydrate were purchased from Sigma-Aldrich and used without any further purification. The synthesis was done in two steps. First, a stoichiometric amount of $Co(CH_3CO_2)_2.4H_2O$ and $Fe(NO_3)_3.9H_2O$ in a molar ratio of 1:2 was dissolved together in 10 ml of 2-methoxyethanol (Sigma Aldrich), then 0.006 mol of ethanolamine (Sigma Aldrich) was added dropwise to the solution (CFO sol). The obtained solution was further stirred for one hour using magnetic stirring. Separately, a stoichiometric amount of $La(NO_3)_3.6H_2O$ and $Fe(NO_3)_3.9H_2O$ in a molar ratio of 1:1 was dissolved in 10 ml of 2-methoxyethanol in another beaker, and 0.25 ml of ethanolamine was added dropwise in the well-dissolved solution (LFO sol). This solution was also kept for stirring for one hour.

After stirring both the solutions for one hour separately, a volumetrically equal amount of both solutions (5 ml of each) were mixed in a beaker. To obtain a thoroughly homogenous LFO-CFO solution, the mixture of LFO-CFO solution was continuous stirred for another one hour. Subsequently, this final homogenous LFO-CFO solution, with 50:50 composition, was used for synthesizing the LFO-CFO composite films via spin coating.

Spin coating details: A freshly cleaned 2 cm × 2 cm high-quality Si/SiO_2-wafer was first treated with oxygen plasma for any surface contamination/cleaning. Afterward, a 150 µL LFO/CFO solution was spin-coated at a spin speed of 3000 rpm for 30 seconds. To dry the film and evaporate any remaining solvent, the spin-coated films were pre-annealed in a hotplate at 200°C for 5 minutes. Finally, to obtain the pure phase and crystalline LFO-CFO films, the spin-coated films were annealed at 800°C for one hour with a heating rate of 20°C/minute, in a high temperate furnace with ambient airflow.

It should be noted that the precursors and solvents used for synthesizing continuous thin films using the simultaneous biphasic synthesis method as described above are different from the ones used for synthesizing particulate nanocomposites [1]. The most critical difference lies in the use of 2-methoxyethanol that allows the synthesis of continuous films, while for particulate nanocomposites, glycine is used as a chelating agent, which also promotes self-combustion of the precursors.

Nanowire fabrication process

The composite nanowires (with widths of 100 nm and 500 nm, and an aspect ratio of 10:1) were fabricated using an electron beam lithography (EBL) process. For the EBL patterning, a Nanobeam Ltd nB5 EBL system was used at an accelerating voltage of 80 kV, with a beam current of 4 nA.

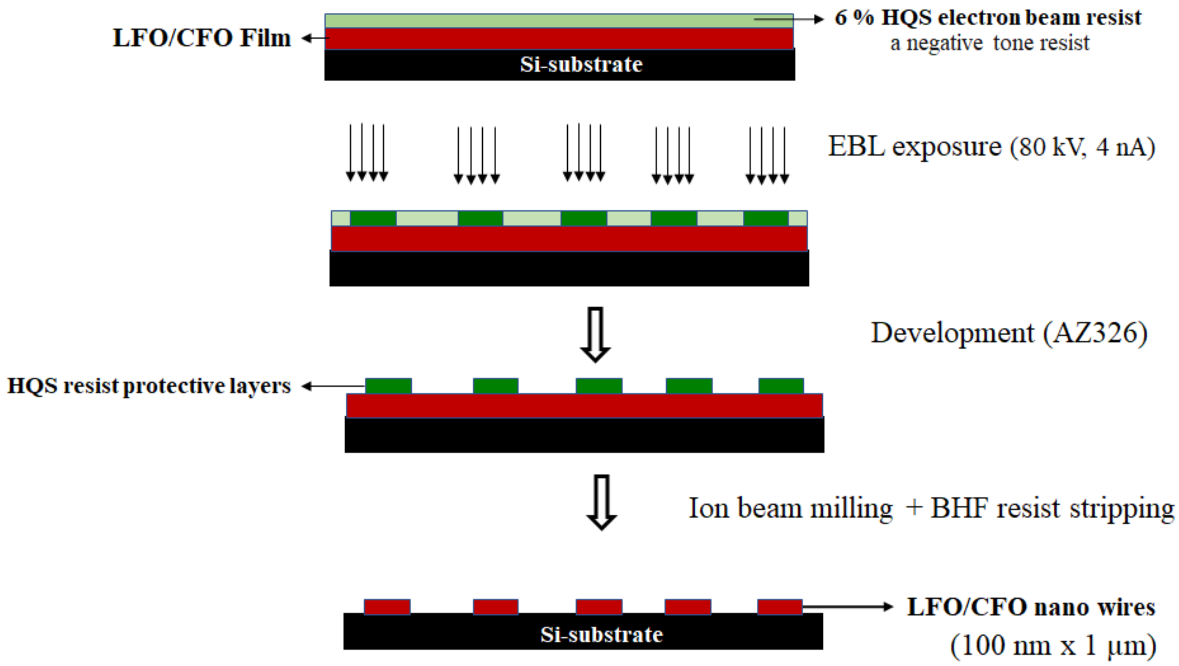

Fig. S1. Schematic showing the EBL process for patterning LFO-CFO composite nanowires on LFO-CFO composite thin films (cross-sectional view).

The EBL process involved the following steps: first, a 130 nm-thick film of 6% HSQ, a negative tone resist for electron beam, was spin-coated on top of the LFO-CFO composite film at a rotation frequency of 6000 rpm for one minute followed by baking at 180°C for 2 min. Next, the composite films with HSQ-resist were patterned by exposing them to an electron beam at 80 kV with a charge dose of 350 $\mu C/cm^2$ in an EBL chamber. The exposed samples were then developed in a solution of AZ-326 developer for 45 seconds and then rinsed in DI water for 1 minute, followed by nitrogen drying and annealing at 180°C for 2 min. During the EBL process, the electron irradiation of the HSQ causes the change in its chemical bonding due to which the solubility of HSQ in the exposed area changes significantly, and it hardens the HSQ in the exposed area. During the developing process, the developer removes all the HSQ resist from the unexposed area, while HSQ over the exposed area remains, which acts as a mask during the ion beam milling.

Ion beam milling, in a Scientific Vacuum Systems i6000, at 500 V, was used to remove the material from the unexposed area of the films and obtain the desired nanowire patterns. During the ion beam milling, the layer of HSQ resist protects the films in the exposed area, and it acts as a hard mask, while the material in the remaining area gets etched away. Finally, the remaining HSQ resist from the structures was removed via a liquid stripping process by dipping the patterned films in 7:1 buffered HF solution (7 parts 40% NH 4F and 1 part 49% HF, without any further dilution of BHF with DI water) for approximately 9 seconds, followed by rinsing in DI water for one minute.

FESEM micrograph of LFO/CFO nanowires over large area

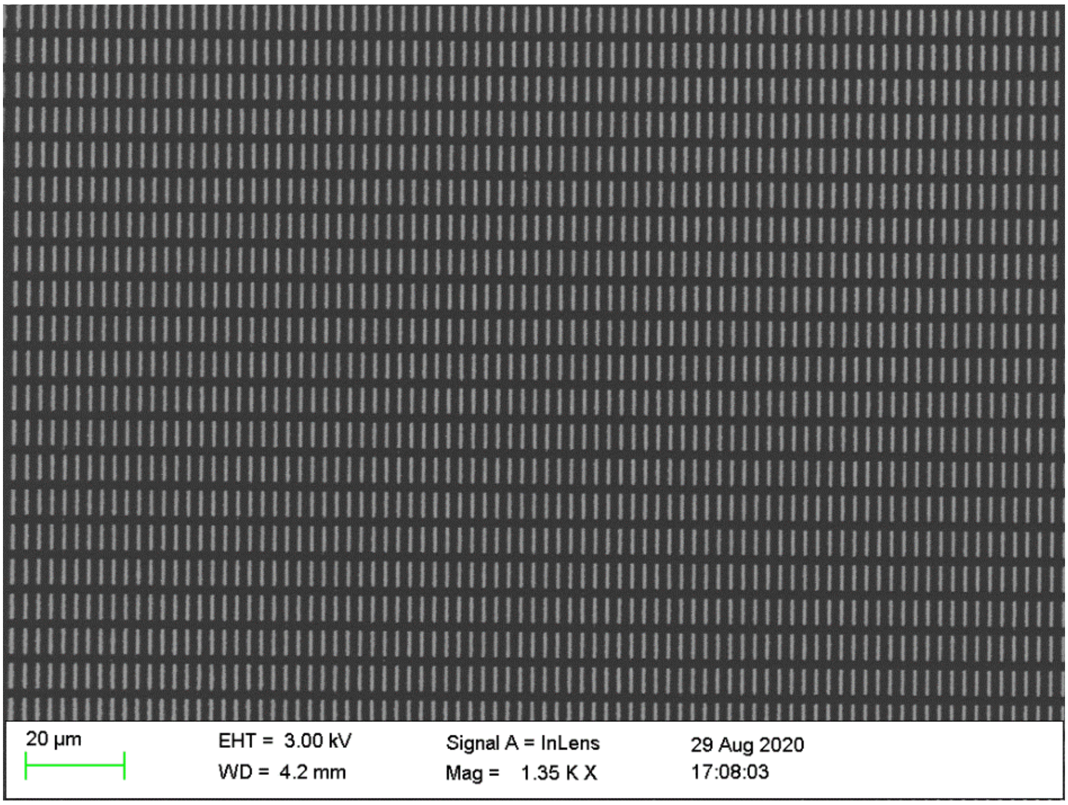

Fig. S2. FESEM micrograph of 500 nm-width LFO-CFO composite nanowires over a large patterned area.

Rietveld refinements on X-ray diffraction pattern of LFO-CFO composite film

To check whether there is any inter-diffusion of the cations, we performed Rietveld analysis on the X-ray diffraction pattern of LFO-CFO composite film using three models – (a) two-phase modeling using LFO and CFO phases with no inter-diffusion, (b) two-phase modeling using LFO and CFO phases with La ion inter-diffused in the CFO structure. The La-content in the CFO structure was kept as a free parameter during refinement, and (c) two-phase modeling using LFO and CFO phases with La ion inter-diffused in the CFO structure. The La-content in the CFO structure was kept fixed at 10% during refinement. We chose La as the diffusing ion since the much larger ionic radius of La^{3+} should cause the maximum effect on the X-ray diffraction patterns. In addition, a theoretical study of cation diffusion in ferrites [2] has suggested that cations of a given oxidation state lying to the left of Fe in the periodic table have lower activation energies for diffusion in ferrites, since they have less number of d-electrons that need to be promoted to high energy t_{2g} orbitals during hopping. In contrast, cations lying to the right of Fe in the periodic table have more d-electrons that need to be promoted to high energy t_{2g} orbitals during hopping, and thus, possess higher barriers to hopping. This suggests that the diffusion activation energy for La-cations should be lower than that of Co-cations, and hence, the probability of diffusion of La-cation into the CFO structure should be higher than that of the diffusion of Co-cation into the LFO structure. Fig. S3 shows the Rietveld fits for the three cases. As is clear from the figure, qualitatively there is no discernible difference between the three fits. Therefore, we have looked at the goodness of fit parameters (σ and R_{wp}), also reported in the respective panels in Fig. S3. We find that the best parameters (smallest values of σ and R_{wp}) are obtained for model (a) i.e., without any inter-diffusion of the cations. For model (b), the La-content in the CFO structure (that was kept as a free parameter during refinement) converged from the starting value of 25% to 5% with a large error of 8%, and the goodness of fit parameters were found to deteriorate slightly compared to case (a). For model (c), where the La-content that diffuses into the CFO structure was kept fixed and not allowed to vary during the refinement, the goodness of fit parameters deteriorate even further. Comparing the results of the three models, we thus find that model (a) with no inter-diffusion of cations gives the best goodness of fit parameters, and is reasonably the most correct. Thus, our analysis suggests that there is no appreciable inter-diffusion of the cations into the bulk of the nanocrystals.

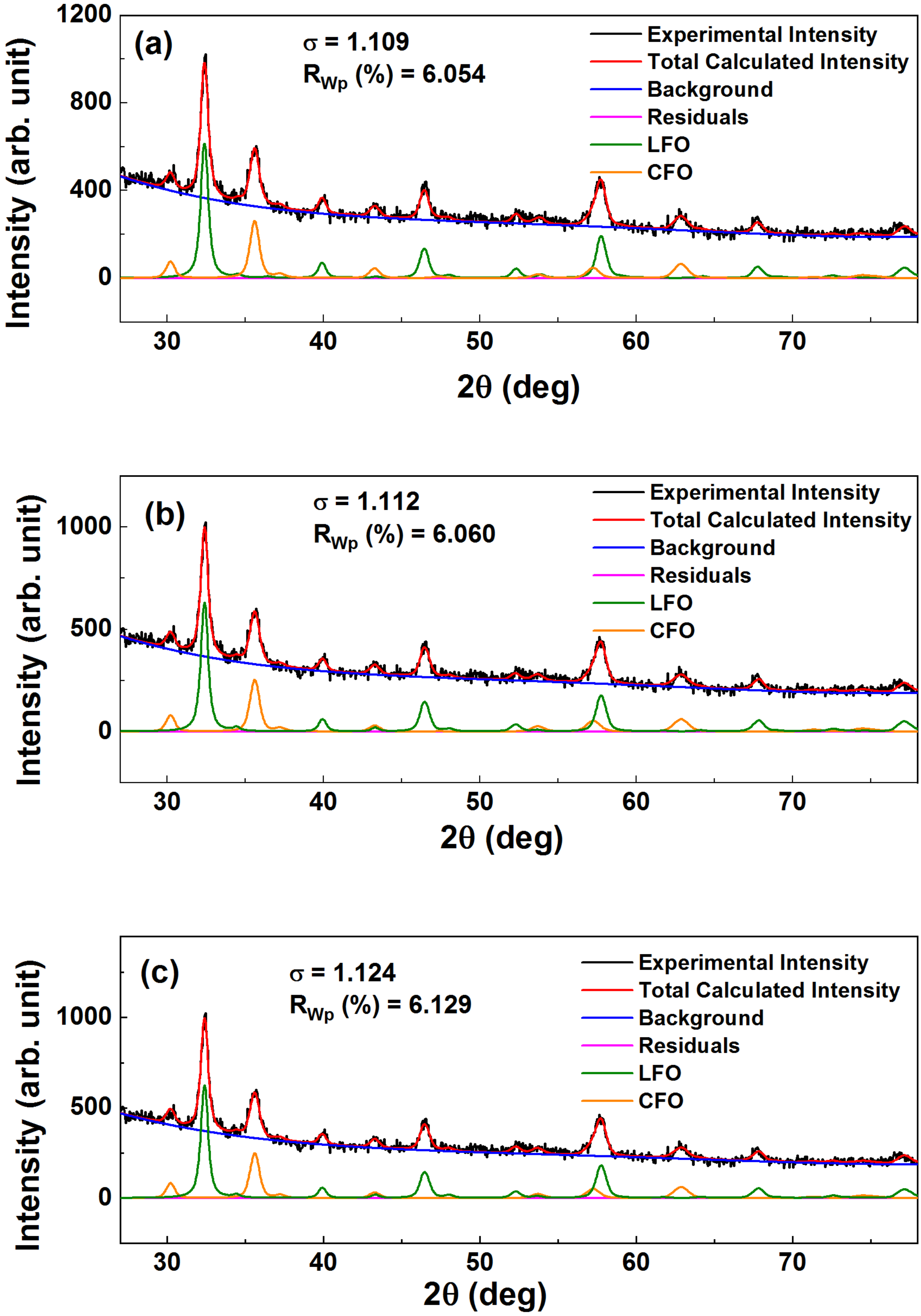

Fig. S3. XRD patterns of LFO-CFO film along with the Rietveld refinement fits and residues using a (a) simple model with no inter-diffusion of the cations, (b) considering La-diffusion into the CFO structure with the La-content as a free parameter during refinement, and (c) considering La-diffusion into the CFO structure with the La-content fixed at 10% during refinement.

Isothermal magnetization curves of LFO-CFO composite film

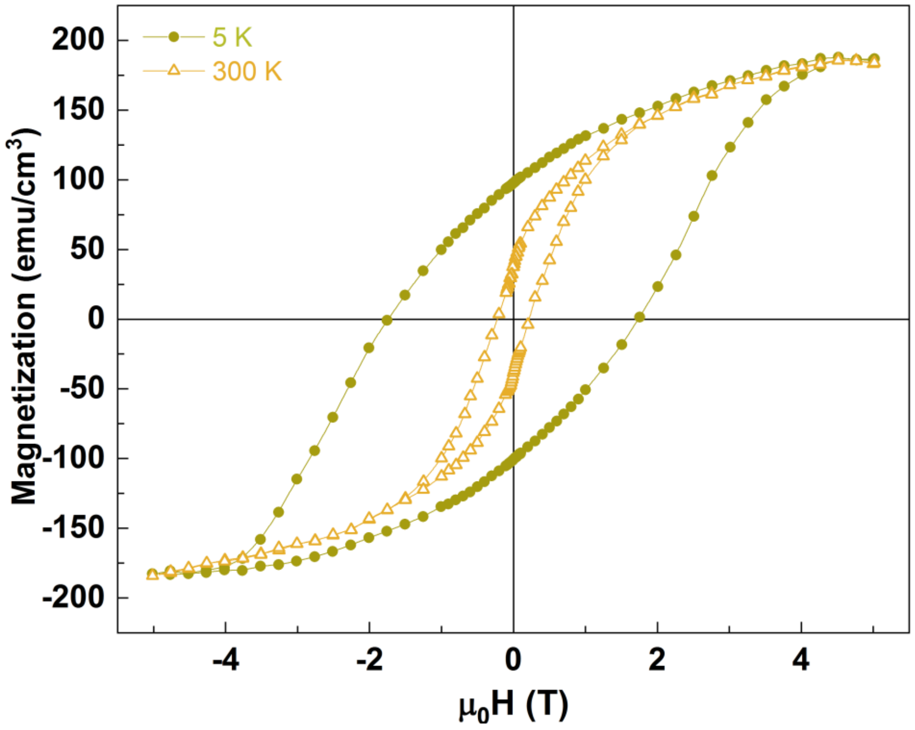

Fig. S4. Isothermal magnetization curves at T = 5 K and 300 K for LFO-CFO composite thin film.

Exchange bias experiments

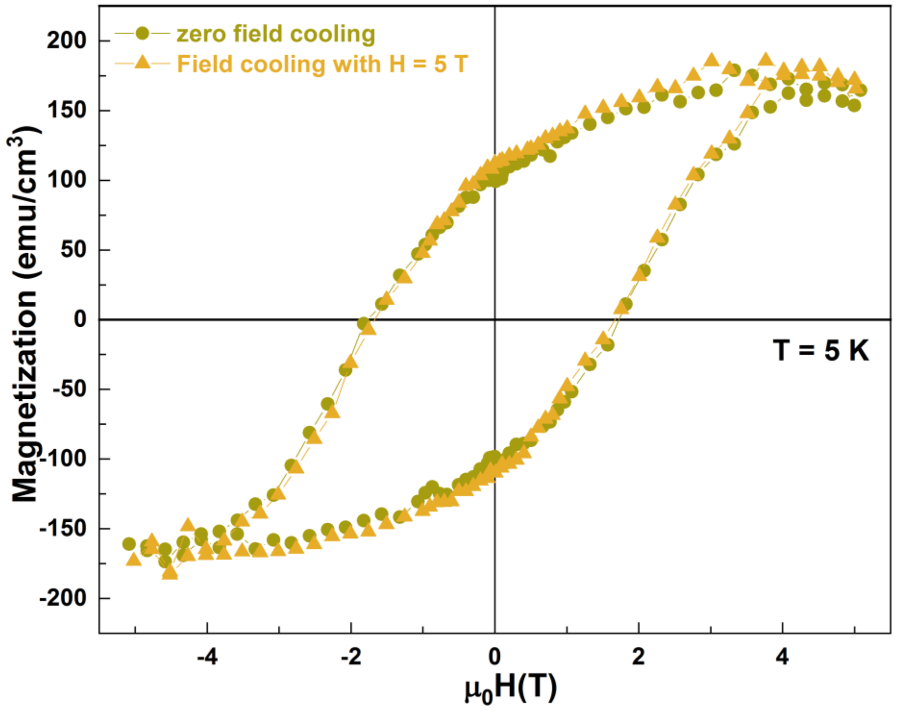

Fig. S5. Isothermal magnetization curves of LFO-CFO composite nanowire of width 500 nm, recorded at T = 5 K after zero field cooling and field cooling from 400 K under a field of 5 T. The sample did not show any exchange bias.

Isothermal magnetization curve of LFO nanocrystals

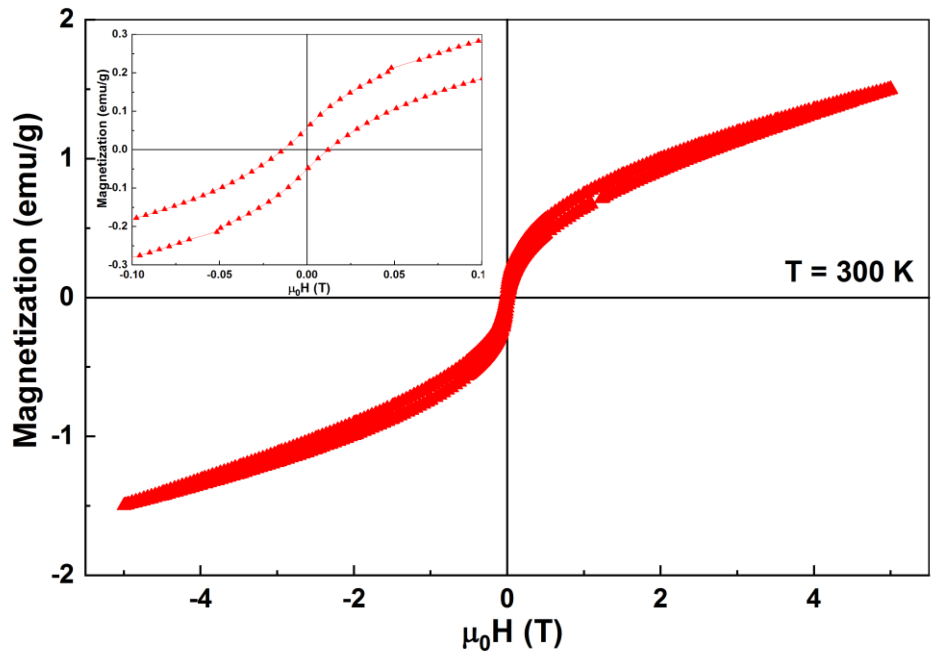

Fig. S6. Isothermal magnetization curve of LFO nanocrystals at T = 300 K. The inset shows the expanded region around low field values.

Weakly coupled LFO-CFO particulate nanocomposites

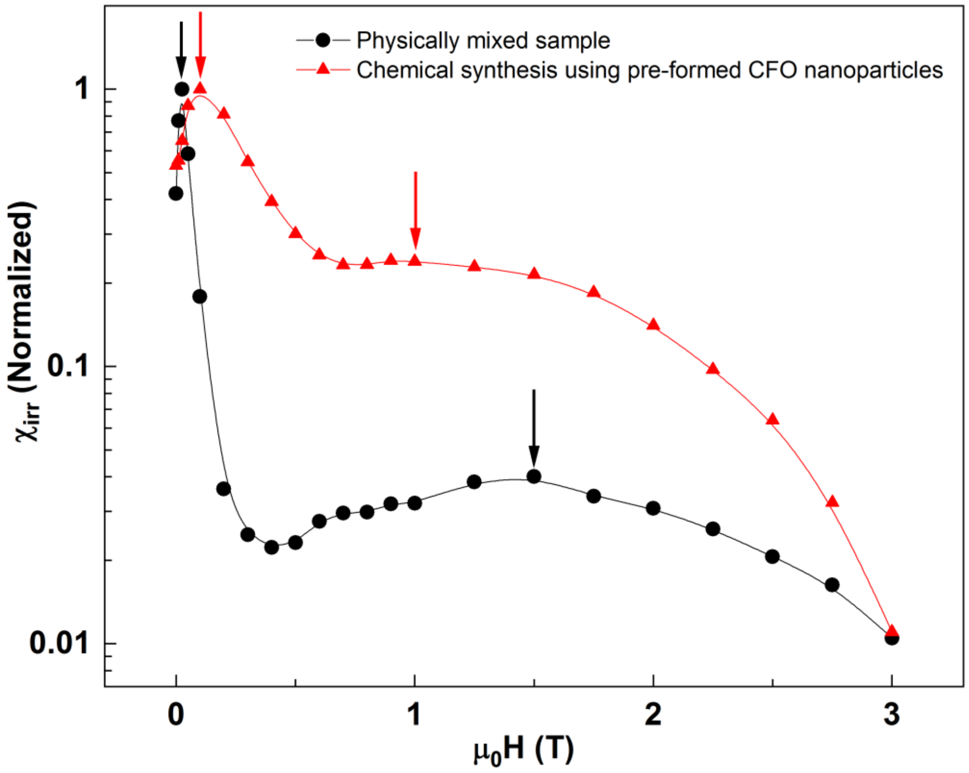

Fig. S7. Switching field distributions at T = 5 K of LFO-CFO particulate nanocomposites with weak magnetic coupling between the two phases.

The χ_{irr} plots in Fig. S7 are for two LFO-CFO particulate nanocomposites synthesized via physical mixing of the two pre-formed phases (black circles) and by chemically synthesizing the LFO phase around pre-formed CFO nanoparticles (red triangles). Both the plots show two distinct peaks corresponding to the reversal processes of the two individual phases, the soft LFO phase that reverses at a very low field (< 0.1 T) and the hard CFO phase with a reversal field > 1 T. In the sample that was prepared by physically mixing the two pre-formed phases (black circles), the coupling is weaker with the two reversal fields farther apart from each other than in the sample where the LFO phase was chemically synthesized around pre-formed CFO nanoparticles (red triangles), where the two reversal fields are closer to each other, indicating that the reversal of one phase partially affects the reversal of the other phase. Nevertheless, the fact that two peaks can be clearly distinguished indicates insufficient coupling between the two phases in both the samples.

References

[1] Sayed, F.; Kotnana, G.; Muscas, G.; Locardi, F.; Comite, A.; Varvaro, G.; Peddis, D.; Barucca, G.; Mathieu, R.; Sarkar, T. Symbiotic, Low-Temperature, and Scalable Synthesis of Bi-Magnetic Complex Oxide Nanocomposites. *Nanoscale Adv.* **2020**, *2* (2), 851–859. https://doi.org/10.1039/c9na00619b.

[2] Muhich, C. L.; Aston, V. J.; Trottier, R. M.; Weimer, A. W.; Musgrave, C. B. First-Principles Analysis of Cation Diffusion in Mixed Metal Ferrite Spinels, *Chem. Mater*. **2016**, *28*, 214–226. https://doi.org/10.1021/acs.chemmater.5b03911.
